# An efficient transformation method for tannin-containing sorghum

**DOI:** 10.7717/peerj.15066

**Published:** 2023-03-14

**Authors:** Yuan Liang, Xuehui Yan, Jingyi Xu, Yanlong Liu, Ke Xie, Jieqin Li, Qiuwen Zhan

**Affiliations:** 1 College of Agricultural, Anhui Science and Technology University, Fengyang, Anhui, China; 2 Research Center of Biology and Agriculture, School of Chemistry and Biological Engineering, University of Science and Technology Beijing, Beijing, China

**Keywords:** Tannin-containing sorghum, Particle bombardment, Genetic transformation, Tissue culture, Transgenic plants

## Abstract

**Background:**

Tannins are the main bottlenecks restricting the transformation efficiency of plants. Hongyingzi is a special tannin-containing sorghum cultivar used in brewing.

**Methods:**

In this study, a highly efficient microprojectile transformation system for tannin-containing sorghum was successfully exploited using immature embryos (IEs) of Hongyingzi as explants.

**Results:**

Hongyingzi presented two types of calli. Type II calli were found to be the most suitable and effective explants for transformation. After optimization of the geneticin (G418) concentration and tissue culture medium, an average transformation frequency of 27% was achieved. Molecular analyzis showed that all transgenic plants were positive and showed transgenes expression. The inheritance analyzis confirmed that the transgenes could be inherited into the next generation. Thus, we successfully established an efficient transformation system for tannin-containing sorghum and demonstrated the possibility of breaking the restriction imposed by tannins in plants.

## Introduction

Sorghum (*Sorghum bicolor* L.) is the fifth most important cereal crop worldwide (Belide et al., 2017), and it shows strong photosynthetic efficiency, a short growth period, and strong stress resistance. Therefore, sorghum plays an important role in ensuring the food supply in drought-prone and saline-alkali soil areas (Gilbert, 2009).

As an important multifunctional crop, sorghum is also an important material for brewing, especially in China (Rooney, Blumenthal & Bean, 2007; Khazaeian, Ashori & Dizaj, 2015; Garzón & Drago, 2018). Ancient Chinese people initially became skilled at the use of sorghum in brewing liquor (Zheng & Han, 2016; McGovern et al., 2004). At present, approximately 80% of Chinese domestic sorghum is used in brewing (Chen et al., 2019). Hongyingzi is an elite variety for brewing because it has great brewing characteristics (Zhou et al., 2020). Over the past 10 years, the cumulative promotional area for Hongyingzi cultivation in China has exceeded 6.7 × 10^6^ hectares and its annual demand has reached approximately six million tons (Wei & Tu, 2020).

Sorghum is widely considered a major recalcitrant crop to tissue culture and transformation (Zhao et al., 2000; Lowe et al., 2009). At present, the efficient genetic transformation of sorghum has significantly improved (Liu & Godwin, 2012; Liu et al., 2020). The highest transformation efficiency by particle bombardment is 60% (Che et al., 2022), while that of agrobacterium-mediated transformation is 30% (Shridhar et al., 2010). However, efficient transformation is based on the non-tannin genotype Tx430; thus, transformation protocols cannot be applied to other tannin-containing sorghum varieties because tannin is the main bottleneck restricting transgenic development (Kuriyama, Shimada & Matsui, 2019; Cai & Butler, 1990; Zhou et al., 2020). Sorghum varieties used for brewing require tannins because this component is the main source of brewing flavour. Therefore, we established an efficient genetic transformation system for tannin-containing sorghum varieties to facilitate the breeding of suitable cultivars for brewing.

In this study, we optimized the tissue culture system and developed a highly efficient transformation protocol using immature embryos (IEs) as explants. We also analyzed the molecular characteristics of T_0_ and T_1_ transgenic plants to confirm both the presence and inheritance of the introduced transgenic genes.

## Materials and Methods

### Plant materials and embryo isolation

Hongyingzi was provided by the Guizhou Drought Grain Sorghum Research Institute and planted in the experimental field of the Anhui University of Science and Technology. Immature seeds were harvested 10–12 days after flowering and surface sterilized using 75% ethanol (v/v) shaking at 220 rpm for 5 min. Subsequently, these immature seeds were transferred to 4% (v/v) sodium hypochlorite supplemented with two drops of Tween 20 and shaken for 10 min. Finally, the immature seeds were washed 5–7 times with sterilized water. IEs were isolated from immature seeds and transferred to a callus induction medium with the scutellum facing up.

### Media for tissue culture

All media were based on MS medium were contained 4.43 g/L M519 powder with vitamins (PhytoTechnology Laboratories, Lenexa, KS, USA) and 30 g/L sucrose, and they were autoclaved at 121 °C for 15 min. Indole-3-butyric acid (IBA), indole-3-acetic acid (IAA), copper sulphate (CuSO_4_), 6-benzylaminopurine (6BA), and 1-naphthaleneacetic acid (NAA) were sterilized using 0.22 µm polyether sulfone (PES) syringe filters. These plant hormones were added after autoclaving at 70 °C. The compositions of different media were modified from previous work (Do et al., 2016; Liu & Godwin, 2012) and are listed in Table 1.

**Table 1:** Media used for transformation experiments.

Medium	Function	Composition (1 L)	pH
MS	Basal medium	4.43 g M519 with vitamins, 30 g sucrose	
CIM	Callus induction	MS supplemented with 1 g potassium dihydrogen phosphate (KH_2_PO_4_), 1 g asparagine, 1 g l-proline, 1 mg 2,4-dichlorophenoxyacetic acid (2,4-D), 0.16 mg (CuSO_4_), 3.5 g Phytagel	5.7
OM	Osmotic medium	MS supplemented with 36.43 g sorbitol, 36.43 g mannitol, 3.5 g Phytagel	5.7
REM	Regeneration	MS supplemented with 1 mg BAP, 1 mg IAA, 0.16 mg CuSO_4_, 3.5 g Phytagel	5.7
SEL-REG	Selective regeneration	MS supplemented with 1 mg 6BA, 1 mg IAA, 0.16 mg CuSO_4_, 50 mg G418, 3.5 g Phytagel	5.7
NRM	New-root induction	MS supplemented with 0.5 g manufacturing execution system (MES),1 mg IBA, 0.1056 mg CuSO_4_, 30 mg G418, 8 g agar	5.8
RM	Root induction	MS supplemented with 1 mg NAA, 1 mg IBA, 0.16 mg CuSO_4_, 30 mg G418, 3.5 g Phytagel	5.7

### Optimization of rooting medium

All transgenic plants were separately transferred to root induction (RM) and new-root induction (NRM) and kept for 10 days in a tissue culture room, in which the temperature was maintained at 28 °C under a lighting regime of 16 h light (100–150 µmol m^−2^ s^−1^) and 8 h dark. The number of roots of the seedlings was recorded and analyzed.

### Constructs

**Figure 1:**
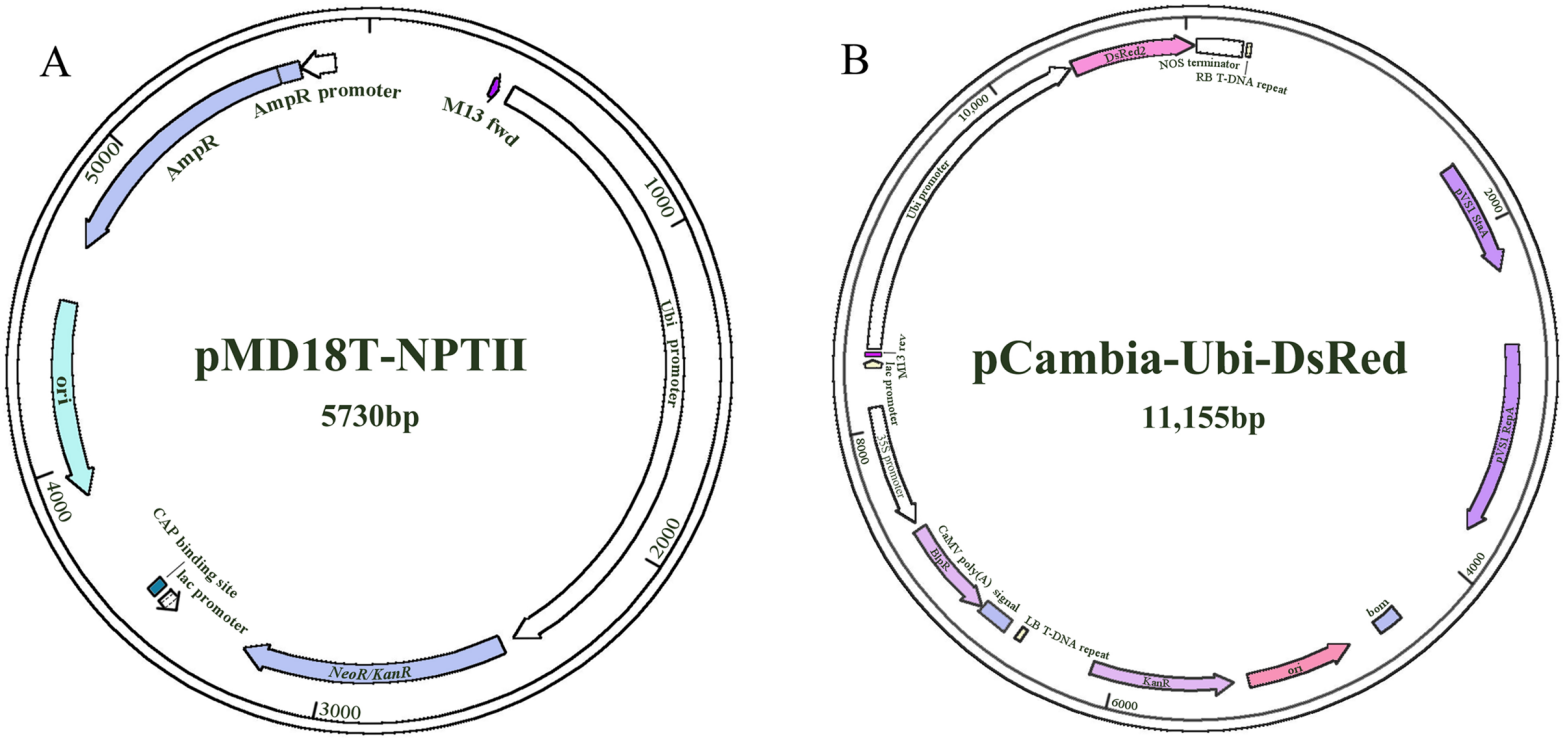
The plasmid maps used for transformation. (A) pMD18T-*NPTII*. (B) pCambia-*Ubi-DsRed*.

The two constructs (*NPTII* and *DsRed*) were provided by the Key Laboratory of Forage Breeding and Utilization of the Anhui Higher Education Institute (Fig. 1). *NPT II* (pMD18T-UBI-NPT II) and *DsRed* (pCambia-Ubi-DsRed) were driven by the maize ubiquitin1 (ubi1) promoter.

### Kill curve experimentation

IEs were incubated on callus induction (CIM) for 9 days. A kill curve experiment was conducted using calli derived from the IEs. The calli were placed on selective regeneration medium containing G418 at 0, 10, 20, 30, 40, and 50 mg/L. Four weeks later, the number of shoots and surviving IEs was recorded and photographed.

### Transformation by particle bombardment

Calli (10–12 per plate) were transferred to the osmotic medium (OM), stored for 2–3 h, and then bombarded. The transgenic protocol was performed as described previously by Wang et al. (2021), with 0.6 gold particles and 9 cm distance from the stopping screen to the calli.

### Selection of transgenic plants

IEs were incubated on CIM for 7–10 days, followed by incubation on SEL-REG dishes for 4–6 weeks. IEs were transferred to fresh medium every 2 weeks. When the callus produced fibrous shoots with a length of 3–6 cm, it was transferred to a tube (150 mm in length, 30 mm in diameter) with a selection NRM medium. After 2 weeks, the transgenic plants were planted in pots with TS1 soil (KLASMANN, Geeste, Germany) and watered once per day. Before being transferred to the greenhouse, they were maintained in a culture room (24–25 °C) for 3 days.

Callus induction, osmosis, and resting were performed in the dark at 28 °C. Selective regeneration and rooting were performed in a tissue culture room at 28 °C under 16 h lighting (100–150 µmol m^−2^ s^−1^) and 8 h dark.

### Molecular analysis of transformants

DNA (DeoxyriboNucleic Acid) was extracted from the leaves of putative transgenic and non-transgenic plants. To confirm the presence of *nptII* and *DsRed*, the fragments *nptII* and *bar* were amplified from genomic DNA using the following specific primer pairs: NPTIINN-f: TCCGGTGCCCTGAATGAA, NPTIINN-r: GTCGATGAATCCAGAAAAGC, and Bar-F: CTCGAGTCTACCA TGAGCCCAGAAC and Bar-R: CTCGAGTCAAATCTCGGTGACGGGCA. The amplified products were 400 and 466 bp, respectively. PCR (polymerase chain reaction) was performed in 30 µL reaction mixtures, which each contained 6 µL of template DNA (50 ng/µL), 4 µL Taq Buffer (20 mM Mg^2+^) 4 µL Taq dNTP (2.0 mM), 6 µL primer (20 mM), and 1.5 µL Taq DNA polymerase (Monad Biolabs). PCR was performed at 94 °C for 7 min, followed by 35 cycles consisting of 94 °C for 30 s, 58 °C for 30 s, 72 °C for 50 s, and a final 7 min elongation step at 72 °C. PCR products were analyzed by electrophoresis using 1.0% agarose gels.

### Reporter gene observation

The pCambia*-Ubi-DsRed* construct was transformed into calli. To detect fluorescence, the roots of putative transgenic and non-transgenic plants were observed and photographed under a Leica DM3000 microscope (Leica, Wetzlar, Germany). Subsequently, the cells of putative transgenic plants were separated from the roots and observed under a microscope.

### Transgene inheritance assay

Seeds of the transgenic plants were harvested for the inheritance assay. Non-transgenic seeds were used as controls. They were placed in a petri dish and soaked in 50 mg/L G418. The dishes were then kept in a tissue culture room and cultured for 1 week under 16 h light and 8 h dark. Photographs were then taken of the transgenic and non-transgenic seeds.

### Statistical analyses

Rooting efficiency date were analyzed using the *t*-test by the SPSS 26.0 statistical software (IBM Corp. Armonk, NY, USA). Triplicate measurements were averaged and included the standard error (±SE).

## Results

### Morphological analyses of calli

**Figure 2:**
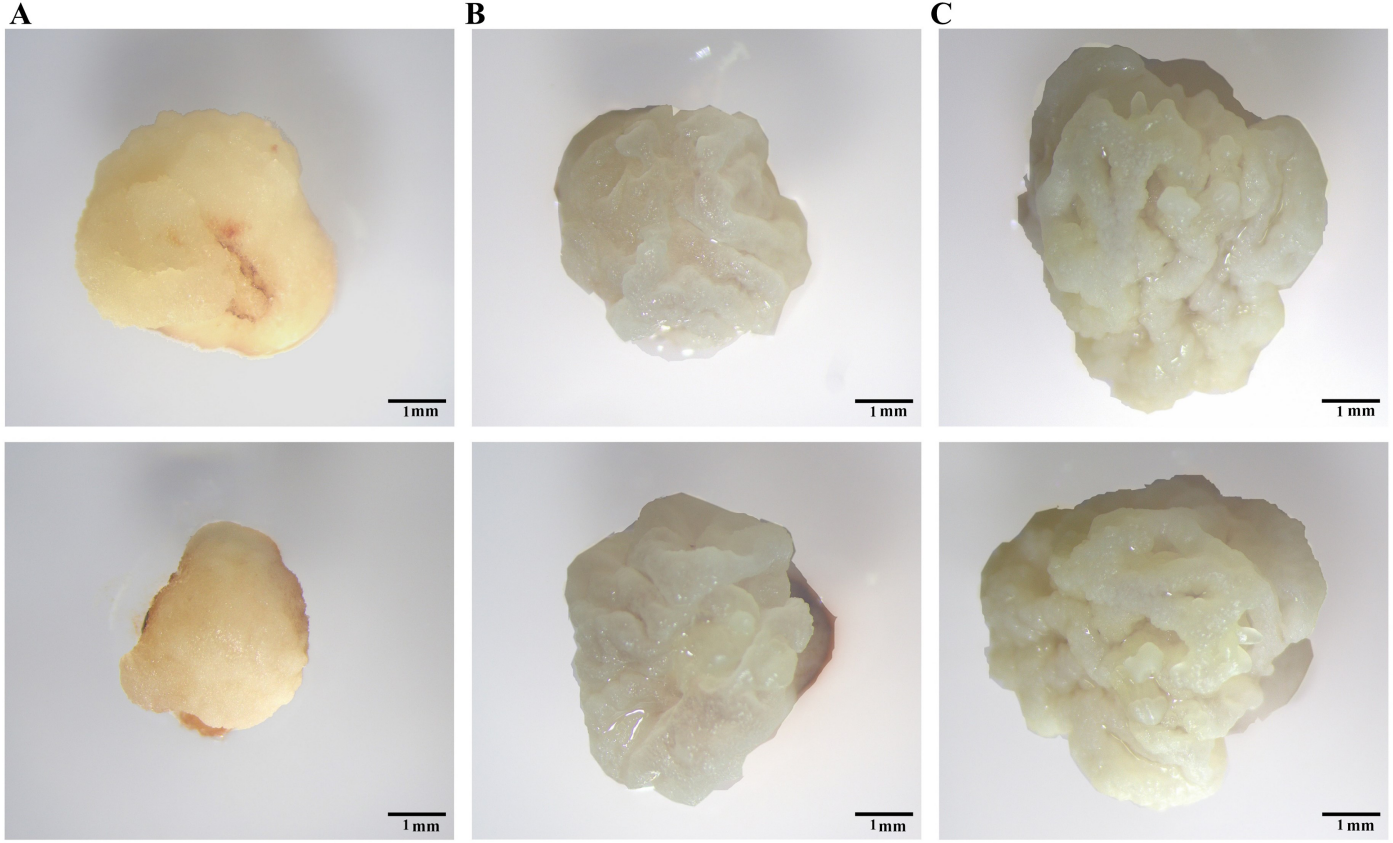
Different calli observed under a YUESHI stereomicroscope. (A, B) 7-day-old callus under the microscope (10 mm). (C) 9-day-old callus under the microscope (10 mm).

Du et al. (2019) identified Type I, Tense Type II, and Loose Type II calli in maize. We discovered two types of calli after induction of IEs for 7 days. Type I calli showed severe browning with a smooth surface and a hard shell (Fig. 2A). Type II calli showed a bright colour, granular shape, and noticeable moisture content (Fig. 2B). Compared to Type I calli, Type II calli grew faster. After 9 days of culture, Type II calli formed a loose structure with a white dry surface (Fig. 2C), which was similar to that of the Loose Type II calli found in maize. In this study, we selected Type II calli for the subsequent research.

### Optimization of rooting medium

**Figure 3:**
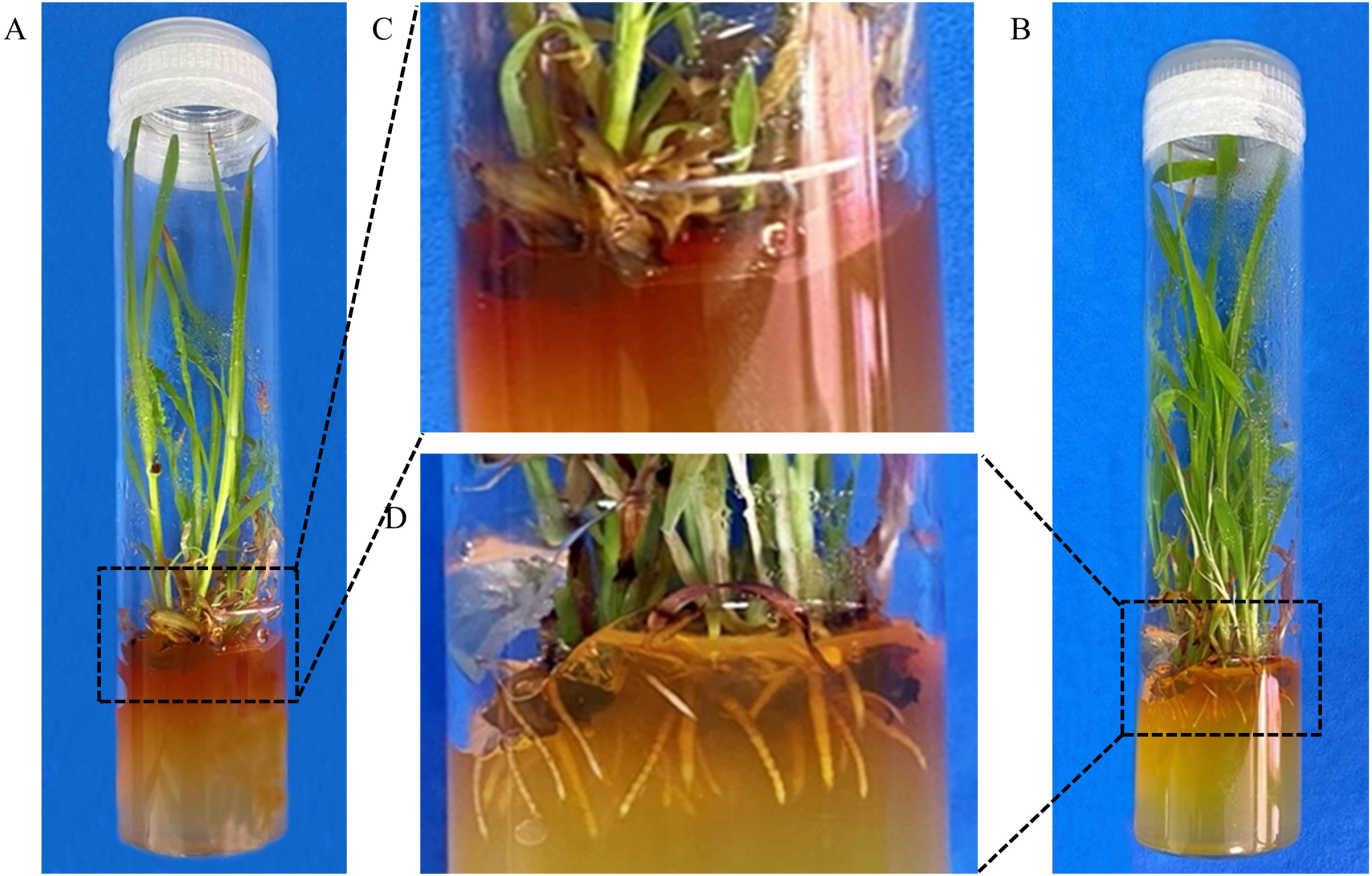
Optimisation of the rooting medium. (A) Plant in RM medium. (B) Plant in NRM medium. (C) Enlarged view of rooting regions of explant shown in A. (D) Enlarged view of roots in B.

To optimize the rooting medium, the rooting efficiency were compared in two different rooting media. In RM, plants did not generate roots and exhibited severe browning (Figs. 3A and 3C). However, the plants could grow roots normally and browning was apparently relieved in NRM (Figs. 3B and 3D). Finally, the average of rooting efficiency was 20.7% ± 2.8% and 64.7% ± 4.7% in RM and NRM, respectively. The results showed that rooting efficiency was significantly higher in NRM than in RM (*P* = 0.00335). Therefore, NRM was used for subsequent transformations.

### Kill curve experiment using G418 on IEs

To optimize the kill concentration for transformation, the different concentrations of G418 were compared (Table 2). The results showed that no IEs or shoots survived at the G50. However, IEs and shoots on regeneration medium showed healthy growth with vigorous shoots at G0 (Table 2). Therefore, G50 was selected for the subsequent transformation experiments.

**Table 2:** G418 kill curve data for Hongyingzi immature embryos (IEs) placed under light for 4 weeks.

Total number	Selective concentration	G418 (mg/L)	IE survival	IE with shoots
15	G0	0	14	14
15	G10	10	10	5
15	G20	20	3	2
15	G30	30	1	0
15	G40	40	1	0
15	G50	50	0	0

### Selection of putative transgenic plants

**Figure 4:**
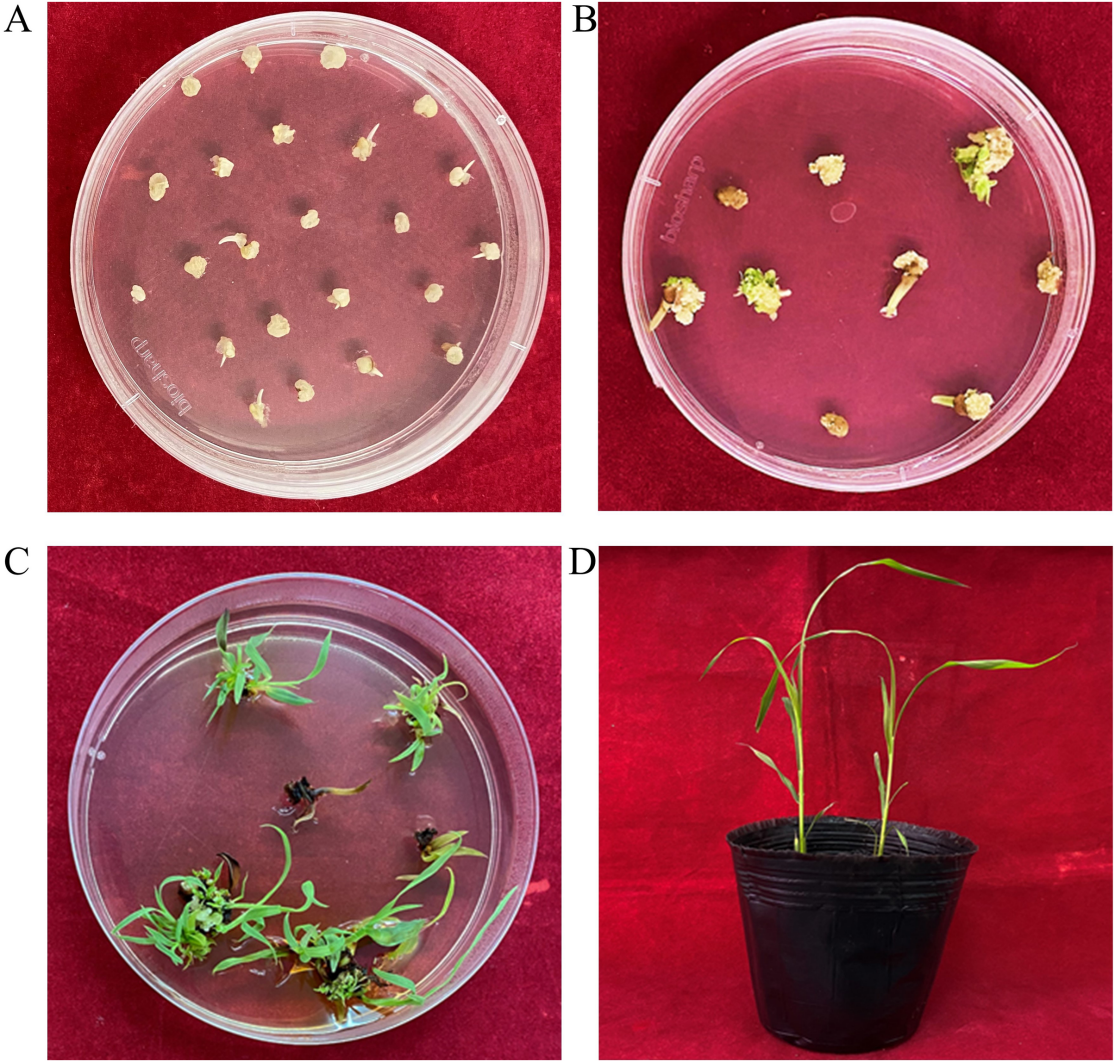
Transformation of Hongyingzi immature embryo. (A) Immature embryos incubated on callus induction medium for 5 days. (B) 14 days after bombardment. (C) Shoots on selection and regeneration medium. (D) Transferred transgenic plants in the pot.

IEs formed small white calli after they were cultured on CIM for 5 days (Fig. 4A). Type II calli were selected for transformation. Necrotic calli and tender leaves appeared in the first 2 weeks on SEL-REG medium (Fig. 4B). In the next 2 weeks, part of the callus formed fibrous shoots (Fig. 4C). Subsequently, these plants were transferred to the NRM for 2 weeks. Plantlets showing strong growth were transplanted into pots for 1 week (Fig. 4D). Then they were planted in a greenhouse. The survival rate was 90%. Approximately 8–11 weeks is required from IEs isolation to planting. The transformation efficiency varied from 25.7% to 28.2%, with an average efficiency of 27% (Table 3).

**Table 3:** Transformation efficiency with IEs of Hongyingzi in three independent experiments.

Experiment	Bombarded IE	Survival number after SEL-REG	Transgenic events	Transformation efficiency (%)
1	35	12	9	25.7
2	46	17	13	28.2
3	44	14	12	27.2
Total	125	43	34	27

### PCR analysis of transgenic events

**Figure 5:**
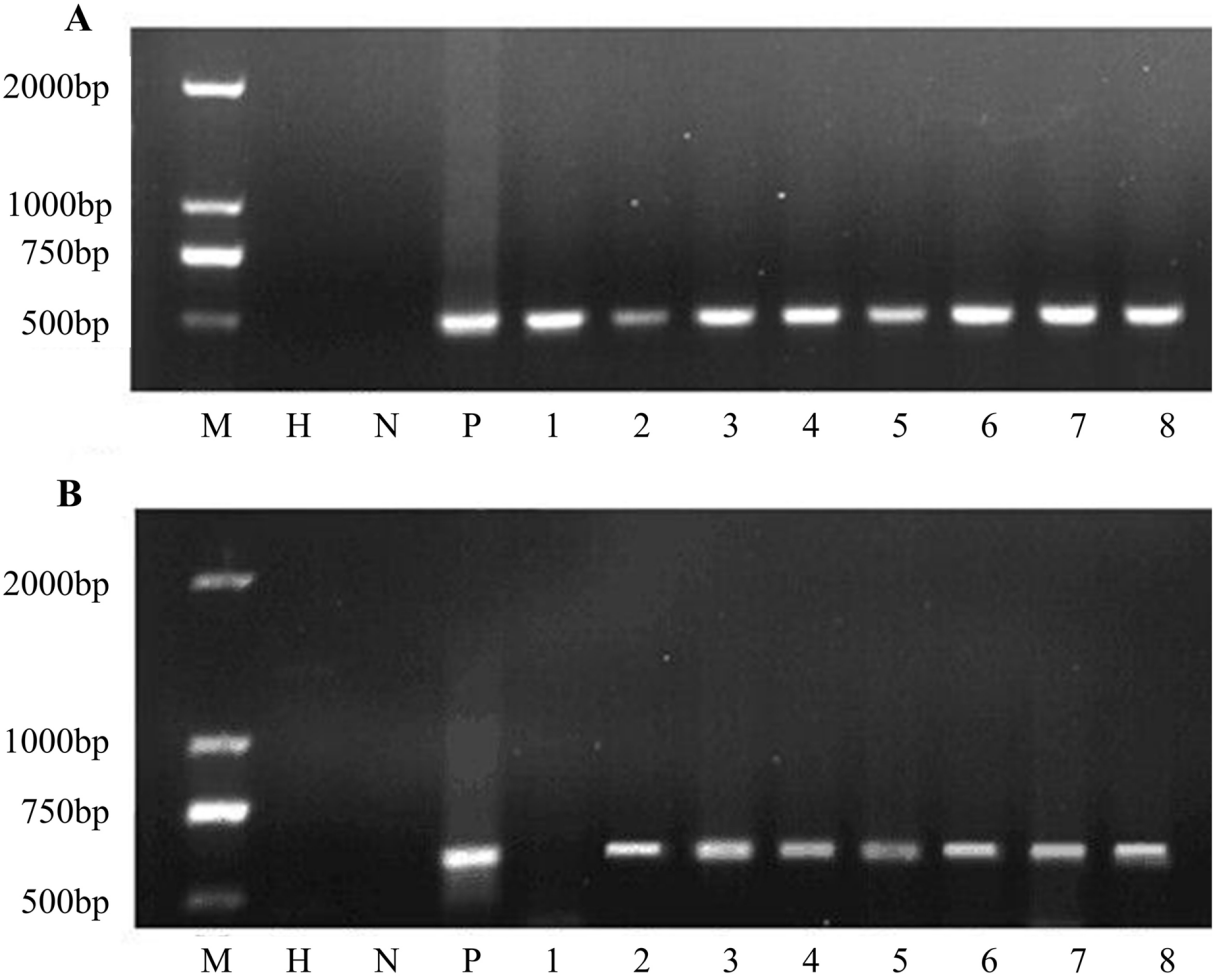
PCR amplification of *nptII* and *bar* genes. (A) PCR screening for *nptII* gene and (B) *bar* gene. M D2000 (Tiangen, Beijing, China). H water, N non-transgenic Hongyingzi, P plasmid, 1–8 eight samples of putative transgenic lines.

Putative transgenic plants were detected using PCR. The results showed that all transgenic plants amplified the same 466 bp fragment as in the construct. Water and non-transgenic plants were used as controls with no PCR products (Fig. 5A). All eight plants were transgenic. Furthermore, the *bar* gene was used for PCR amplification. PCR analysis revealed that seven of the eight samples showed the *bar* gene specific fragment (Fig. 5B). In general, the co-transformation rate was 87.5%.

### Red fluorescent protein expression

**Figure 6:**
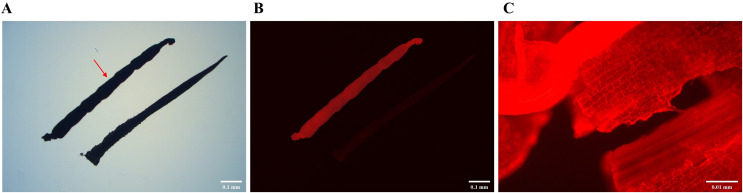
Red fluorescent protein expression under the green light of fluorescence microscope. (A) Transgenic root (with red arrow) and non-transgenic root under white light. (B) Roots under green light. (C) The root cell of transgenic plant under 40× fluorescence microscope.

To verify whether the *DsRed* gene was properly expressed in transgenic plants, the roots from transgenic and non-transgenic plants were observed. The root from transgenic plants showed a bright red colour, whereas the roots from non-transgenic plants did not (Figs. 6A and 6B). The bright red colour also can be observed from root cells in transgenic plants (Fig. 6C). The results confirmed that the *DsRed* gene was expressed normally in transformed sorghum.

### Transgene inheritance assay

**Figure 7:**
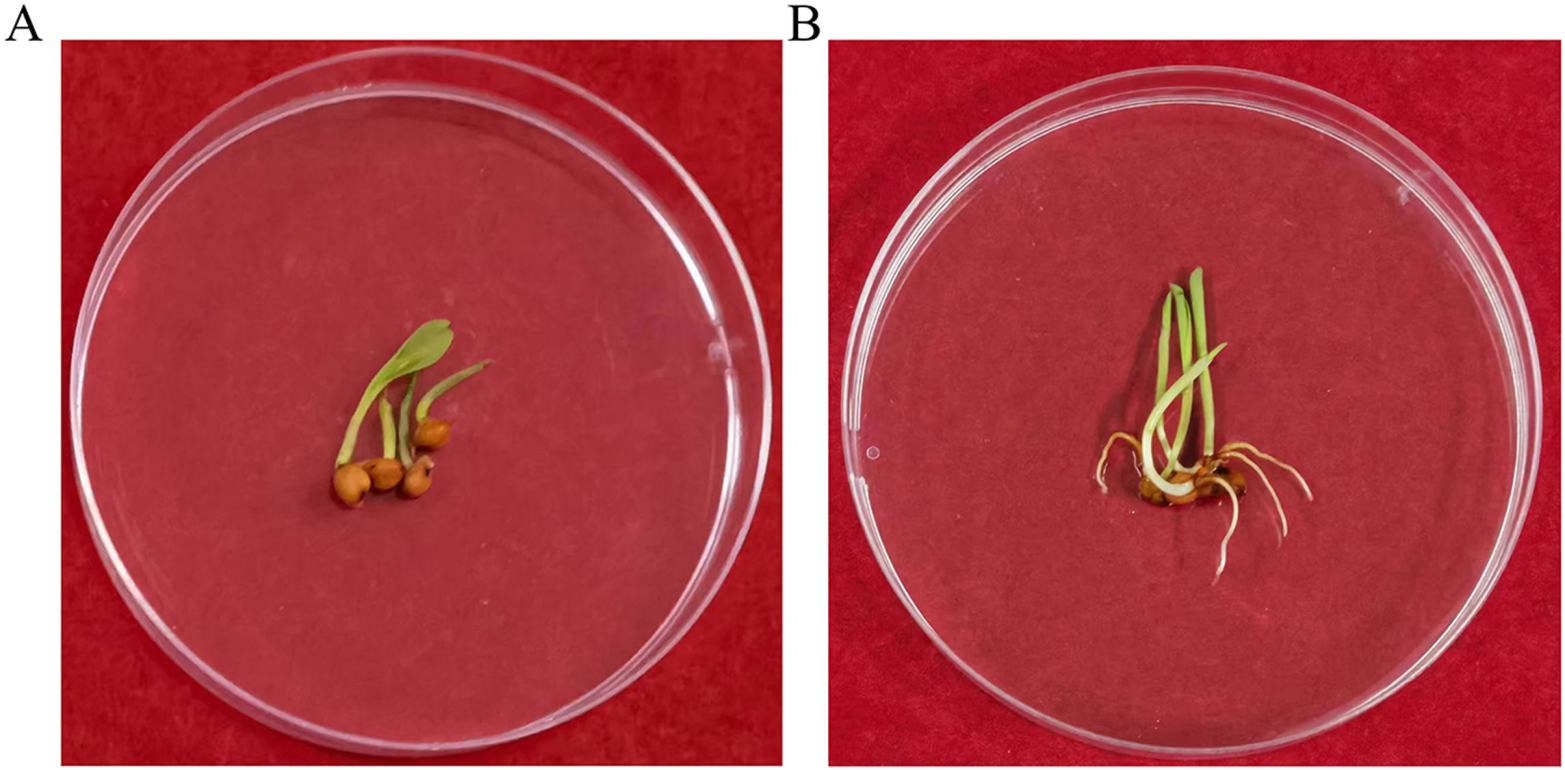
The G418 resistant assay. (A) The non-transgenic seedlings. (B) The transgenic T_1_ seedlings.

**Figure 8:**
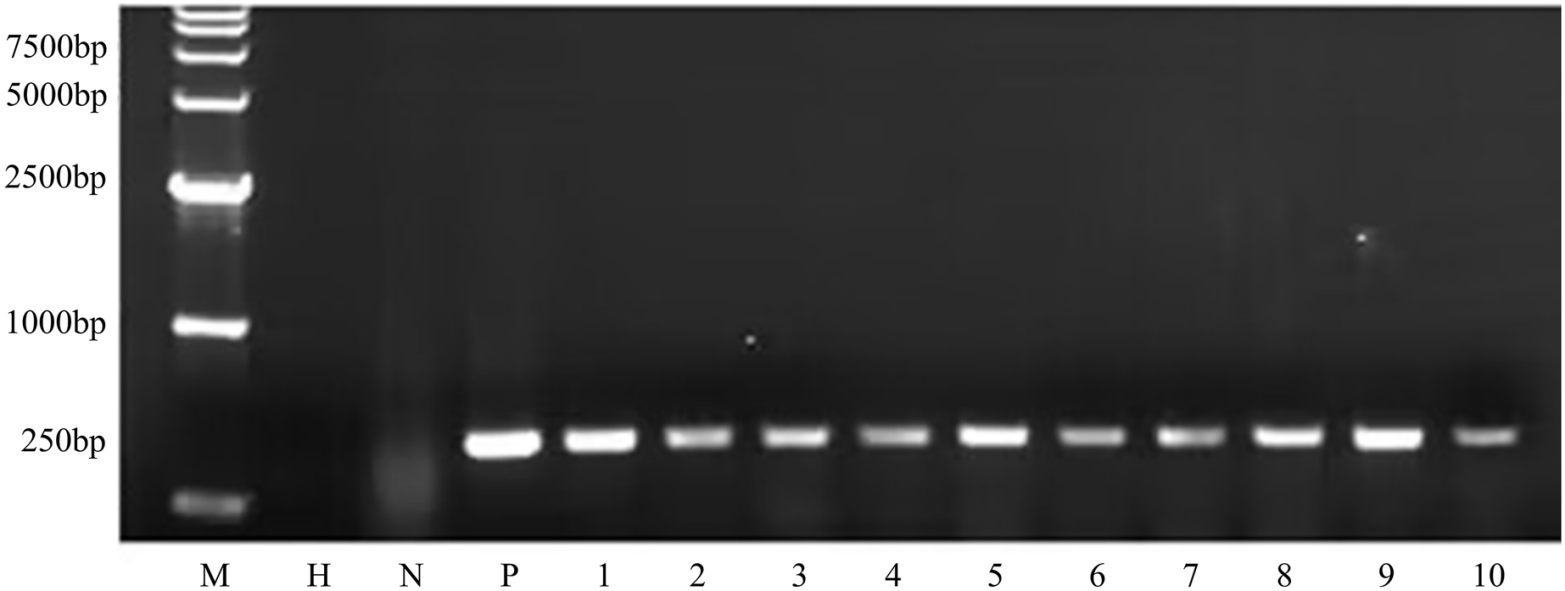
PCR amplicons of *bar* gene in T_1_ generation. M D15000 (Tiangen, Beijing, China). H water, N non-transgenic Hongyingzi, P plasmid, 1–10 samples of transgenic T_1_ lines.

To determine whether the transgene *nptII* was inherited and expressed in the next generation, an inheritance assay was performed for non-transgenic and transgenic seeds. The results showed that non-transgenic seeds did not grow roots in the G418 solution (Fig. 7A). However, putatively transformed plants grew normally in G418 solution (Fig. 7B). PCR also was performed to analyze the T_1_ generation of transgenic plants. The results showed that all transgenic plants amplified the same fragment as the *bar* plasmid at 466 bp. The water and non-transgenic plants were not able to amplify any fragments (Fig. 8). These results showed that the transgene was stably inherited into the next generation.

## Discussion

Sorghum grains possess tannins, which are phenolic compounds (Chen et al., 2021). These compounds are ubiquitous in plants and represent the most widely distributed secondary metabolites (Xu, Wang & Zhao, 2021). Presently, the highest genetic transformation efficiency for non-tannin-containing sorghum is 60.9% (Che et al., 2022). In this research, the average transgenic efficiency was 27%. Although we optimized the medium, the transgenic efficiency was still lower than that in previous research. Tannins likely represent main reason for the lack of improvement in transgenic efficiency. Kuriyama, Shimada & Matsui (2019) demonstrated that phenolic compounds are considered the main constraint in the efficient transformation of sorghum explants (Cai & Butler, 1990; Dykes et al., 2005; Dykes & Rooney, 2006; Kuriyama, Shimada & Matsui, 2019). In addition, tannins also negatively affect callus growth and quality (Dykes & Rooney, 2006). Rooting is an important step in transformation. In this study, we compared the rooting efficiency of two rooting media; the results showed that the rooting efficiency significantly differed between the media. The difference was mainly due to the effects of different phytohormones on the production of phenolic compounds in the medium. In the present study, we adjusted the contents of NAA and MES in the rooting medium, based on the findings of a study by Do et al. (2016) that NAA and MES affected rooting efficiency during sorghum transformation. Subsequently, the rooting efficiency significantly improved. Furthermore, our study demonstrated similar result, *i.e*., the contents of NAA and MES affected rooting efficiency because they may have interacted with other hormones to promote cell division, thereby promoting rooting efficiency.

High tannin content is the main reason sorghum has become a raw material for brewing renowned liquor, and sorghum varieties with 1–2% of tannins are used as raw material for brewing Moutai-flavour liquor (Zhou et al., 2020). Tannins promote the formation of catechuic acid, vanillin, and other substances and provide a special flavour for Baijiu, a Chinese liquor (Wei et al., 2020). Furthermore, tannins in sorghum can reduce bird predation during seed maturation (Xie et al., 2019). Therefore, establishing a genetic transformation system for tannin-containing sorghum is beneficial. In this study, we established a highly efficient transformation system for tannin-containing sorghum, which will also serve as a potential reference for research regarding other tannin-containing plants.

## Conclusions

This study established a highly efficient transformation system for sorghum, which is considered difficult to transform owing to its high tannin content. The system used IEs of Hongyingzi as explants. An average transgenic efficiency of 27% was achieved in the Hongyingzi plants after optimisation of the G418 concentration and tissue culture medium. Only one cultivated species was examined in this study. We believe that our study makes a significant contribution to the literature because it supports the effective genetic transformation of not only sorghum but other plants with low transformation efficiency caused by high tannin contents. Moreover, this study could serve as a potential reference for further research in relevant fields of transgenics and crop improvement.

## Supplemental Information

10.7717/peerj.15066/supp-1 Supplemental Information 1 Date processing.

10.7717/peerj.15066/supp-2 Supplemental Information 2 PCR amplification of *npt II* gene. A *npt II* gene .M D2000 (Tiangen, China). H water, N non-transgenic Hongyingzi, P plasmid, 1–8 8 samples of putative transgenic lines.

10.7717/peerj.15066/supp-3 Supplemental Information 3 The data of rooting efficiency.

10.7717/peerj.15066/supp-4 Supplemental Information 4 PCR amplification of *bar* gene. B *bar* gene. M D2000 (Tiangen, China). H water, N non-transgenic Hongyingzi, P plasmid, 1–8 8 samples of putative transgenic lines.

10.7717/peerj.15066/supp-5 Supplemental Information 5 PCR amplicons of *bar* gene in T_1_ generation. M D15000 (Tiangen, China). H water, N non-transgenic Hongyingzi, P plasmid, 1–10 samples of transgenic T_1_ lines.

10.7717/peerj.15066/supp-6 Supplemental Information 6 Original blot for Figure 5.

## Additional Information and Declarations

### Data Availability

The following information was supplied regarding data availability: The raw measurements are available in the Supplemental Files.
