# Supplementary figures and images for: An efficient transformation method for tannin-containing sorghum
Source: PeerJ. 2023 Mar 14;11:e15066. doi: 10.7717/peerj.15066 (PMC10022505; doi:10.7717/peerj.15066)

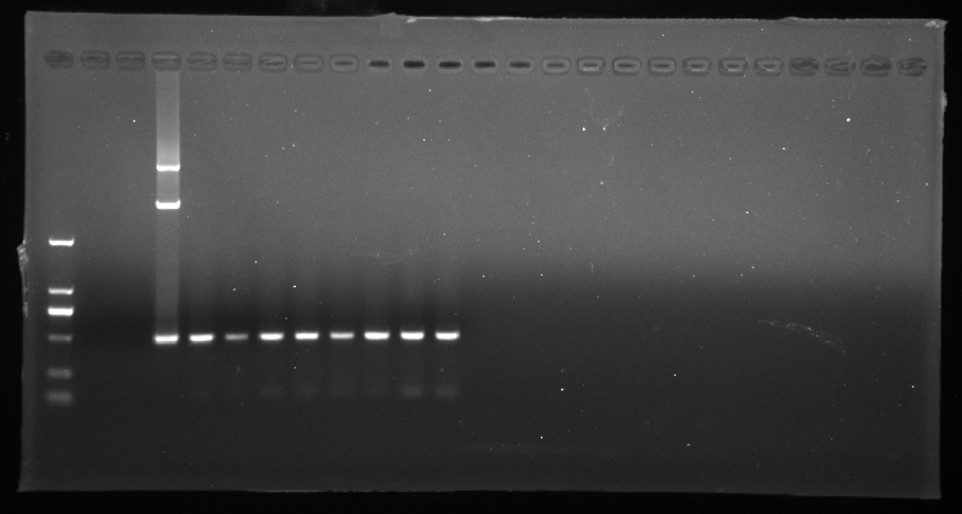

Supplement: Supplemental Information 2 — A npt II gene .M D2000 (Tiangen, China). H water, N non-transgenic Hongyingzi, P plasmid, 1–8 8 samples of putative transgenic lines. [file peerj-11-15066-s002.jpg]

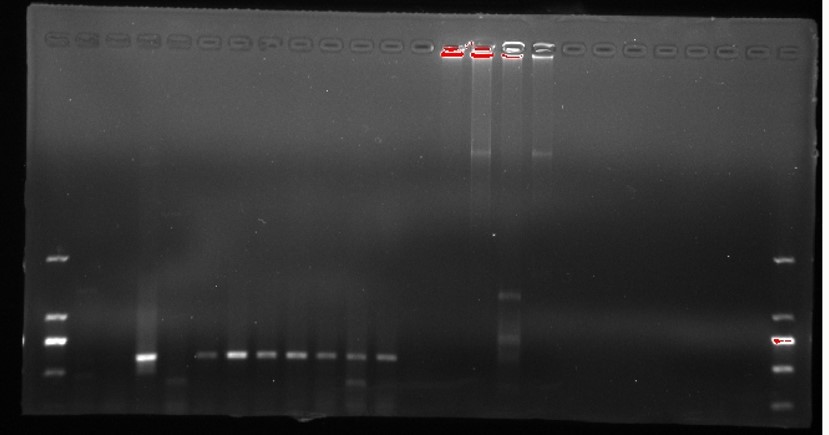

Supplement: Supplemental Information 4 — B bar gene. M D2000 (Tiangen, China). H water, N non-transgenic Hongyingzi, P plasmid, 1–8 8 samples of putative transgenic lines. [file peerj-11-15066-s004.jpg]

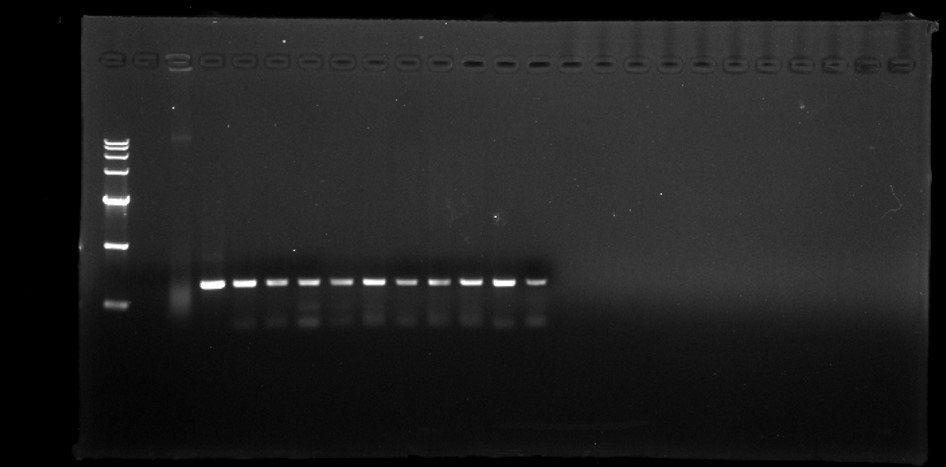

Supplement: Supplemental Information 5 — M D15000 (Tiangen, China). H water, N non-transgenic Hongyingzi, P plasmid, 1–10 samples of transgenic T1 lines. [file peerj-11-15066-s005.jpg]
