# Supplementary material for: An efficient transformation method for tannin-containing sorghum
Source: PeerJ. 2023 Mar 14;11:e15066. doi: 10.7717/peerj.15066 (PMC10022505; doi:10.7717/peerj.15066)

**The left part of the full-length uncropped gel was used in our research article.**

**The part on the right was not relevant to this experiment and article.**


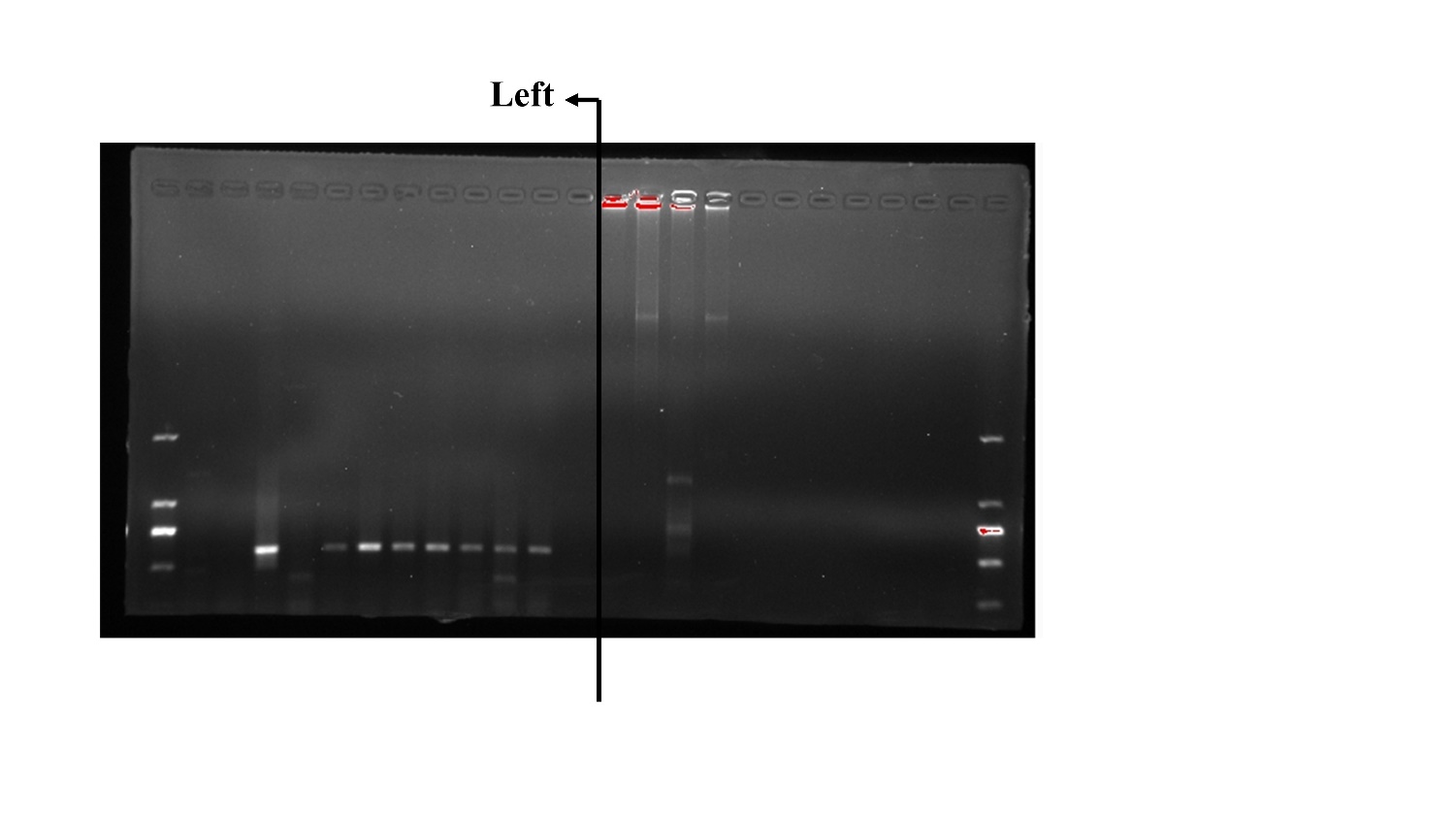

Supplement: Supplemental Information 6 [file peerj-11-15066-s006.docx]
